# Compassionate Use of Cefiderocol to Treat a Case of Prosthetic Joint Infection Due to Extensively Drug-Resistant *Enterobacter hormaechei*

**DOI:** 10.3390/microorganisms8081236

**Published:** 2020-08-13

**Authors:** Soline Siméon, Laurent Dortet, Frédérique Bouchand, Anne-Laure Roux, Rémy A. Bonnin, Clara Duran, Jean-Winoc Decousser, Simon Bessis, Benjamin Davido, Grégory Sorriaux, Aurélien Dinh

**Affiliations:** 1 Infectious Disease Unit, Raymond-Poincaré Hospital, AP-HP, Paris Saclay University, 92380 Garches, France; soline.simeon@aphp.fr (S.S.); clara.drn@gmail.com (C.D.); simon.bessis@aphp.fr (S.B.); benjamin.davido@aphp.fr (B.D.); 2 Microbiology laboratory, Bicêtre Hospital, AP-HP, UMR 1184, Team Resist, INSERM, Paris-Saclay University, Faculty of Medicine, French National Reference Center for Antibiotic Resistance: Carbapenemase producing Enterobacteriaceae, 94270 Le Kremlin-Bicêtre, France; laurent.dortet@aphp.fr (L.D.); remy.bonnin@u-psud.fr (R.A.B.); 3 Pharmacy, Raymond-Poincaré Hospital, AP-HP, Paris Saclay University, 92380 Garches, France; frederique.bouchand@aphp.fr; 4 Microbiology laboratory, Raymond-Poincaré Hospital, AP-HP, Paris Saclay University, 92380 Garches, France; anne-laure.roux@aphp.fr; 5 Department of Bacteriology and Infection Control, Henri Mondor University Hospital, AP-HP, 94000 Créteil, France; jean-winoc.decousser@aphp.fr; 6 EA 7380 Dynamyc, University Paris-Est Créteil (UPEC), Ecole nationale vétérinaire d’Alfort (EnvA), Faculty of Medecine, 94000 Créteil, France; 7 Surgery department, Jouvenet Clinique, 75016 Paris, France; docteur.sorriaux@orange.fr

**Keywords:** cefiderocol, prosthetic joint infection, antibiotic, bacterial resistance

## Abstract

We report the case of a 67-year old man with a right knee prosthetic joint infection due to extensively drug-resistant *Enterobacter hormaechei*. The resistance phenotype was due to the overproduction of the intrinsic cephalosporinase (ACT-5) associated with the production of three acquired β-lactamases (CTX-M-15, TEM-1B and OXA-1), and a putative membrane decreased permeability. He was first treated with colistin-tigecyclin due to adverse drug reactions; treatment was switched to cefiderocol for a 12-week antibiotic duration, with a favorable outcome.

## 1. Case Presentation

A 67-year-old man, with several comorbidities (atrial fibrillation on anticoagulant, chronic glaucoma), a previous surgical history of total left knee replacement 5 years before, aortic stent 2 years before for abdominal aortic aneurysm, and a right knee replacement surgery for gonathrosis two and a half months before, presented at an orthopedic surgery department in Jouvenet Clinique in Paris, France, with local tenderness, swelling, red and inflammatory skin, fever with chills, and functional impotence of the right knee for the last 2 weeks, suggesting acute prosthetic joint infection (PJI). The X-ray did not show prosthesis loosening and the CT scan showed no abscess. The patient was hospitalized and underwent surgery. He was managed with DAIR (debridement, antibiotics and implant retention) of the right knee prosthesis. Intraoperative observations found a purulent discharge. Surgery involved prosthesis’ mobile parts exchange and debridement of soft tissue. Empirical antimicrobial therapy with piperacillin-tazobactam (4 g qid) and vancomycin (25 mg/kg/day after loading dose) was initiated immediately post-surgery.

The growth culture of the intra-operative samples revealed an extensively drug-resistant (XDR) *Enterobacter hormaechei*, resistant to carbapenem. In this context, the patient was transferred to an infectious diseases department of Raymond-Poincaré Hospital, which is a French referral center for complex bone and joint infections, five days after surgery. At admission, the patient was afebrile, with pain and inflammatory scaring; his white blood cell count was 8.2 G/L and C-reactive protein (CRP) level was 34.5 mg/L. Blood cultures remained negative. The microbiological and the whole genome analysis of the *Enterobacter hormaechei* subsp. *hoffmanii* strain by the National Reference Centre for Antibiotic Resistance (CNR) showed that the bacteria did not produce any carbapenemase, even though it was resistant to carbapenems. The observed resistance phenotype was due to the overproduction of the intrinsic cephalosporinase (ACT-5) associated with the production of three acquired β-lactamases (the extended-spectrum β-lactamase (ESBL) CTX-M-15, plus two narrow spectrum enzymes, TEM-1B and OXA-1), and decreased putative membrane permeability. On top of β-lactams resistance, whole genome sequencing revealed that this isolate also acquired several genetic determinants responsible for resistance to all aminoglycosides (*aac(3)-IIa*, *aac-(6′)-1b-cr*, *aadA1*, *aph(3”)-IIb*, *aph-(6)-Id*), fluoroquinolones (*aac-(6′)-1b-cr*, *qnrB1*), sulphonamides (*sul2*), trimethoprim (*dfrA14*) and tetracyclin (*tet(A)*). Accordingly, this bacterial isolate remained susceptible only to tigecycline (minimal inhibitory concentration (MIC) = 1 mg/L) and colistin (MIC = 0.5 mg/L) among the antibiotics tested (Table 1).

The patient was switched at Day 7, for intravenous sodium colistimethate 4.5MU bid and tigecycline 100 mg bid, after a loading dose for each antibiotic. One week later, he presented with adverse drug-reactions: nausea, anorexia and renal failure (increase in serum creatinine level of 2 mg/L). Considering the susceptibility testing results and adverse-drug reactions from Colistin and Tigecyclin treatment—cefiderocol, a siderophore cephalosporin developed by Shionogi and Co. (Osaka, Japan)—was tested.

Cefiderocol MIC was 1 mg/L by the microdilution method at French National Reference Center for antibiotic resistance, indicating its susceptibility according to the European Committee on Antimicrobial Susceptibility Testing (EUCAST) breakpoints (Table 1). Intravenous cefiderocol monotherapy of 2 g every 8 h infused over 3 h was administered, considering literature data on pharmacokinetics, pharmacodynamics, and tolerability, with close monitoring of patient’s renal function [1]. The patient was discharged from hospital at Day 21, and continued receiving outpatient parenteral antibiotic therapy. He was treated for a total of 12 weeks of effective antibiotic treatment, as recommended by the usual guidelines, including 10 weeks of cefiderocol monotherapy treatment. Clinical and biological course was favorable, with return to normal physical activity and complete regression of the biological inflammatory syndrome. No adverse drug reaction was observed. Nausea and loss of appetite regressed, and kidney function normalized. At 12 months from the end of the antibiotic treatment, the patient recovered totally with no recurrence.

## 2. Discussion

To our knowledge, this is the first case of PJI due to XDR Enterobacterales successfully treated with cefiderocol monotherapy. Alamarat et al. reported a compassionate use of cefiderocol to treat chronic osteomyelitis caused by metallo-β-lactamase (MBL)-producing *Pseudomonas aeruginosa* and an ESBL-producing *Klebsiella pneumoniae* in a 15-year-old patient [2]. The patient had a successful clinical outcome, with only self-resolved possible hematological toxicity. Zingg et al. recently published two other cases of bone and joint infections treated successfully with cefiderocol: a 29-year-old male patient with acute osteomyelitis of the tibia due to an early postoperative implant-associated polymicrobial infection with carbapenemase-producing *P. aeruginosa* (VIM-type), *Acinetobacter baumannii* (OXA-23), and *Enterobacter cloacae* (KPC-type), and a 64-year-old male patient with an early postoperative implant-associated infection of the spine with *A. baumannii* (NDM, OXA-40) [3].

Currently, a large proportion of carbapenem-resistant infections are caused by Gram-negative bacteria, where resistance is related to porin loss and efflux pump activity, as in our present case [4]. To the extent that some authors suggest that the rate of carbapenem resistance—not due to carbapenemase, especially among non-fermenters—has exceeded Enterobacterales, represents a greater challenge for the treatment of severe infections [4].

Several studies showed in vitro activity of cefiderocol against CR-GNB [5,6]. Regarding CR Enterobacterales, in vitro activity of cefiderocol was more potent than meropenem, ceftazidime-avibactam or ceftolozane-tazobactam, as with our XDR *E. hormaechei* strain [6].

Few clinical cases were also published, describing cefiderocol’s compassionate use for other types of infection, such as endocarditis, ventilator-associated pneumonia, intra-abdominal infection or bloodstream infection [7,8,9].

In these studies, cefiderocol MICs of the tested strains were inferior to the provisional susceptibility breakpoint of ≤4 mg/L used for Enterobacterales, *P. aeruginosa*, *Stenotrophomonas maltophilia* and *Acinetobacter* spp., and proposed by the Clinical & Laboratory Standards Institute (CLSI), based on preclinical pharmacokinetic and pharmacodynamics studies [10].

However, following the approval of cefiderocol in late 2019 by the FDA, alternative breakpoints were adopted for Enterobacterales and *P. aeruginosa* (≤2 and ≤1 mg/L, respectively). Cefiderocol MIC testing must be conducted with iron-depleted, cation-adjusted Mueller-Hinton broths as in our case, despite not being yet commercially available, thus creating uncertainty about the interpretation of testing conditions and results.

To achieve the best outcome for our patient with a difficult-to-treat infection—also likely due to the presence of biofilm—we made the choice of the regimen (2 g q8h, 3-h infusion) based on pharmacokinetics data observed in phase I and II studies, and on the simulation of Katsube et al., incorporating cefiderocol MICs from 1260 clinical isolates of *P. aeruginosa*, *A. baumannii*, and Enterobacterales (from 0.25 to 16 mg/L) [1,11]. In simulated subjects with normal renal function, this regimen achieved ≥  75% *f*T  >  MIC in at least 90% of cases. Although the study found acceptable target attainment with 1-h infusions, the authors supported the 3-h infusion based on the potential use of cefiderocol for severe infections [11].

For our patient, no adverse reaction was observed, although a long treatment duration was prescribed. In cefiderocol trials, some data about adverse drug reactions are available (https://www.fda.gov/media/131703/download). The most frequently reported adverse events were digestive disorders and known adverse effects of cephalosporins, such as *C. difficile*-related diarrhea (https://www.ema.europa.eu/en/documents/assessment-report/fetcroja-epar-public-assessment-report_en.pdf).

Our case highlights the unique challenges in managing patients with biofilm-associated infections such as PJI due to XDR GNB, which is deemed difficult-to-treat. Moreover, the efficacy of cefiderocol against bacterial biofilm development remains unknown.

## 3. Conclusions

PJI due to XDR Gram-negative bacteria constitutes a major therapeutic challenge. Cefiderocol can be a therapeutic option in these cases, considering its tolerability and broad-spectrum activity. In difficult-to-treat infections such as PJI, more data are warranted to assess best dosage regimen and efficacy of cefiderocol.

## Figures and Tables

**Table 1:** Antimicrobial minimal inhibitory concentrations of the *E. hormaechei* subsp. *hoffmanni.*

Antimicrobials	MIC (mg/L) (Clinical Categorization)
	EUCAST Breakpoints ^i^
**β-lactams**	
Amoxicillin	>256 (R)
Amoxicillin + clavulanate ^a^	>256 (R)
Ticarcillin	>256 (R)
Ticarcillin + clavulanate ^a^	64 (R)
Piperacillin	>256 (R)
Piperacillin + tazobactam ^b^	128 (R)
Temocillin	>32 (R)
Cefoxitin	>256 (R)
Aztreonam	>32 (R)
Cefotaxime	>32 (R)
Ceftazidime	>32 (R)
Ceftazidime + avibactam ^c^	8 (S)
Cefepime	>32 (R)
Cefepime + zidebactam ^d^	8
Ceftolozane + tazobactam ^b^	>32 (R)
Ertapenem	>32 (R)
Imipenem	8 (R)
Imipenem + relebactam ^e^	1 (S)
Meropenem	16 (R)
Meropenem + vaborbactam ^f^	8 (S)
Cefiderocol ^g^	1 (S)
**Aminoglycosides**	
Gentamicin	>256 (R)
Tobramycin	48 (R)
Amikacin	16 (R)
**Quinolones**	
Levofloxacine	>32 (R)
Ciprofloxacine	>32 (R)
**Cyclines**	
Tigecycline ^h^	1 (S)
Eravacycline ^h^	2 (S)
**Other**	
Sulfamethoxazole-trimethoprim	>32 (R)
Chlorampenicol	>256 (R)
Colistin	0.5 (S)

MIC: minimal inhibitory concentration; S: Susceptible; I: Intermediate; R: Resistant. ^a^ clavulanate concentration is fixed at 2 mg/L. ^b^ tazobactam concentration is fixed at 4 mg/L. ^c^ avibactam concentration is fixed at 4 mg/L. ^d^ cefepime/zidebactam ratio is (1:1). ^e^ relebactam concentration is fixed at 4 mg/L. Breakpoints of imipenem alone were used for clinical categorization. ^f^ vaborbactam concentration is fixed at 8 mg/L. ^g^ MIC was determined using iron-depleted, cation-adjusted Mueller-Hinton broth. ^h^ Tetracycline breakpoints were used for clinical categorization. ^i^ Clinical categorization was determined using EUCAST breakpoints as updated in 2020 (https://www.eucast.org/fileadmin/src/media/PDFs/EUCAST_files/Breakpoint_tables/v_10.0_Breakpoint_Tables.pdf).
